# Robotic Versus Laparoscopic Cholecystectomy in Complex Acute Cholecystitis: A Comparative Analysis of Postoperative Recovery

**DOI:** 10.7759/cureus.102933

**Published:** 2026-02-03

**Authors:** Biswabasu Das, Sandeep Kumar Sahu, Durga Bhavani, Neelima Priya

**Affiliations:** 1 Surgical Gastroenterology and Robotic Surgery, Care Hospitals, Visakhapatnam, IND

**Keywords:** cholecystectomy, laparoscopic surgery, pain, postoperative recovery, robotic-assisted surgery

## Abstract

Background

Robotic cholecystectomy (RC) has emerged as an alternative to laparoscopic cholecystectomy (LC) in the management of complex acute cholecystitis (AC); however, real-world comparative data on perioperative outcomes and postoperative recovery remain limited. This study aimed to compare short-term perioperative outcomes and recovery parameters following RC and LC for complex AC in routine clinical practice.

Materials and methods

This was a retrospective observational study conducted using prospectively maintained clinical records. Consecutive adult patients diagnosed with complex AC, defined as Grade II or Grade III disease according to the Tokyo Guidelines, who underwent RC or LC between February 2024 and July 2025 were included. All procedures were performed by a single surgeon experienced in both techniques, thereby minimizing operator-related variability.

Results

In total, 142 patients undergoing cholecystectomy were evaluated, of whom 91 underwent an RC and 51 underwent an LC. Baseline clinical profiles were largely balanced between the two cohorts. However, the RC group included a greater proportion of male patients and demonstrated a higher distribution of American Society of Anesthesiologists (ASA) physical status classifications. Conversion to open surgery occurred more frequently in the LC group, whereas no conversions were recorded in the RC group (p<0.001). The mean total port count differed significantly between the groups, with a higher number observed in the RC cohort. Patients in the RC group demonstrated significantly shorter ICU and total hospital stays, as well as earlier resumption of oral intake and independent ambulation (all p-values <0.001). Time to first postoperative bowel movement also occurred earlier in the robotic group, although this difference did not reach statistical significance (p=0.0668). Postoperative pain scores were significantly lower in the RC cohort at 24 hours and at seven, 14, and 30 days following surgery (all p-values <0.001, except day 30; p=0.0042). Patients treated with RC achieved a faster return to routine daily activities, with a statistically significant between-group difference (p<0.001). Intraoperative complication rates were comparable between the groups, and no postoperative complications, 30-day readmissions, or deaths occurred in either cohort.

Conclusion

RC was associated with improved perioperative outcomes and faster postoperative recovery compared with LC in complex AC, without increased short-term morbidity or mortality. Prospective multicenter studies with standardized outcome assessment are required to validate these findings.

## Introduction

Acute cholecystitis (AC) is a potentially life-threatening condition, most commonly resulting from obstruction of the cystic duct by gallstones. It is characterized by localized gallbladder inflammation, right upper quadrant pain, fever, and systemic inflammatory manifestations [1]. Since its introduction in 1985, laparoscopic cholecystectomy (LC) has become the standard of care for the surgical management of cholecystitis, owing to its well-established advantages over open cholecystectomy, including less postoperative pain, reduced intraoperative blood loss, shorter hospitalization, and more rapid recovery of function [2].

In recent years, robotic cholecystectomy (RC) has been increasingly adopted across surgical disciplines, including emergency general surgery, facilitated by enhanced three-dimensional visualization, improved instrument articulation, and superior ergonomic control. Despite these technical advantages, the clinical benefit of RC over LC remains a subject of debate. While several studies have reported favorable perioperative outcomes and improved recovery with RC [3-6], others have demonstrated outcomes comparable to or, in some instances, inferior to those achieved with LC [7-10]. Importantly, much of the existing literature comprises heterogeneous patient populations that include both acute and chronic cholecystitis, thereby limiting disease-specific inferences. Evidence specifically evaluating the role of RC in AC, particularly in complex cases, remains sparse. Within the Indian context, only a single study has directly compared RC and LC in patients with AC [3]. Furthermore, patient-centered recovery outcomes extending beyond the immediate perioperative period have not been adequately characterized. Against this backdrop, the present study was undertaken to compare perioperative outcomes and postoperative recovery in patients with complex AC undergoing RC versus LC in routine clinical practice.

## Materials and methods

Study design and setting

This study comprised a retrospective analysis of a prospectively maintained clinical database conducted at a single tertiary care center, Care Hospitals, Visakhapatnam, India, between February 2024 and July 2025. The study included consecutive adult patients who underwent RC or LC for AC classified as Grade II or Grade III according to the Tokyo Guidelines for the Diagnosis and Severity Grading of AC [11]. Grade II or moderate AC was defined by the presence of any one of the following clinical or laboratory findings: leukocytosis with a white blood cell (WBC) count >18,000/mm³, a palpable and tender mass in the right upper quadrant, symptom duration longer than 72 hours, or evidence of significant local inflammatory changes. Grade III or severe AC was defined by the presence of organ or system dysfunction, including cardiovascular, neurological, respiratory, renal, or hepatic involvement.

Baseline patient and laboratory characteristics data collected included age, sex, body mass index (BMI), American Society of Anesthesiologists (ASA) physical status classification [12], history of prior surgery, and preoperative laboratory values. Intraoperative data collected comprised Tokyo severity grade, operative time, port configuration, and intraoperative complications. Postoperative outcomes assessed included duration of ICU stay, total length of hospital stay, postoperative laboratory values, time to independent ambulation, time to resumption of oral intake, time to first bowel movement, postoperative complications, postoperative pain scores, and time to return to activities of daily living. Any reoperation or hospital readmission occurring within 30 days of the index procedure was recorded. Pain severity after surgery was measured on a 10-point Visual Analogue Scale (VAS) [13], in which zero denoted the absence of pain and 10 corresponded to the most severe pain imaginable. The VAS, a validated and reproducible single-item pain assessment tool, was administered within 24 hours postoperatively and subsequently at one, two, and four weeks following surgery. The study was performed in compliance with the Declaration of Helsinki and relevant Good Clinical Practice guidelines. Approval was granted by the Institutional Ethics Committee (approval no. 02/IECC/2025, dated November 3, 2025). All participants provided general informed consent permitting treatment and the use of de-identified clinical data for research purposes.

All robotic and laparoscopic procedures were performed by a single experienced surgeon using standard multiport techniques recommended by professional surgical societies. The da Vinci X Surgical System (Intuitive Surgical, Sunnyvale, CA, USA) was used for all robotic procedures. The operating surgeon has over three decades of experience in LC and has surpassed the learning curve for RC, and has performed more than 20 robotic cases within the three-month period before the study. Accordingly, the present analysis reflects outcomes achieved beyond the learning curve.

Statistical analysis

All study data were securely stored in MS Excel (Microsoft Corp., Redmond, WA, USA) spreadsheets with restricted access. Numerical variables were described using appropriate measures of central tendency and dispersion, reported either as means with standard deviations or as medians with interquartile ranges. Discrete variables were summarized using absolute counts and proportions. Comparative analyses between study groups were undertaken using chi-square or Fisher’s exact tests for categorical data, while continuous outcomes were evaluated with either independent-samples t-tests or non-parametric Mann-Whitney U tests according to distributional characteristics. Statistical inference was based on two-tailed testing, with significance defined at p<0.05. All computations were performed using R software (version 4.5.0, R Foundation for Statistical Computing, Vienna, Austria).

## Results

The study cohort comprised 142 patients, with 91 undergoing RC and 51 undergoing LC. Overall, the two groups were well matched with respect to baseline patient characteristics, with only minor variations observed. The average age of patients in the RC group was 53.23 ± 13.89 years, compared with 49.44 ± 16.94 years in the LC group. Men accounted for a greater proportion of patients in the RC cohort (68.13%) than in the LC cohort (47.06%), although this difference did not reach statistical significance (p=0.137). Previous abdominal surgery was reported in 21.98% (n=20) of patients in the RC group and 13.33% (n=17) of those in the LC group, with no statistically meaningful difference between cohorts (p=0.1392). With respect to preoperative risk stratification, 10 patients (10.99%) in the RC group had an ASA physical status score of IV, whereas no patients in the LC group were classified as ASA IV (p=0.0137). The distribution of ASA scores also differed significantly between the groups. The distribution of patients according to the Tokyo Grade of AC, as assessed at the surgeon’s discretion, was comparable between the groups (p=0.9266). Preoperative laboratory parameters, including alanine aminotransferase (ALT), aspartate aminotransferase (AST), alkaline phosphatase (ALP), White Blood Cells (WBC) count, and serum bilirubin levels, did not differ significantly between the two groups. Table 1 provides an overview of the baseline patient characteristics.

**Table 1:** Baseline characteristics of the study population * Significant value (p<0.05) RC: Robotic Cholecystectomy; LC: Laparoscopic Cholecystectomy; SD: Standard Deviation; ASA: American Society of Anesthesiologists

Parameter	RC (N=91)	LC (N=51)	t-value/χ^2^	P-value
Age, mean ± SD, years	53.23 ± 13.89	49.44 ± 16.94	1.44	0.1522
Gender, n (%)				
Female	29 (31.87)	27 (52.94)	6.08	0.0137*
Male	62 (68.13)	24 (47.06)	-	-
ASA score, n (%)				
I	30 (32.97)	19 (37.25)	9.61	0.0222*
II	40 (43.96)	19 (37.25)	-	-
III	11 (12.09)	13 (25.49)	-	-
IV	10 (10.99)	0 (0.0)	-	-
Tokyo grade, n (%)				
II	78 (85.71)	44 (86.27)	0.01	0.9266
III	13 (14.29)	7 (13.73)	-	-
Previous abdominal surgery, n (%)	20 (21.98)	17 (13.33)	2.19	0.1392
White blood cells, mean ± SD, 10^3 ^cells/µL	7.43 ± 3.74	6.93 ± 2.83	-0.49	0.6227
Alanine aminotransferase, mean ± SD, U/L	38.00 ± 27.98	40.67 ± 15.20	-1.20	0.2341
Aspartate aminotransferase, mean ± SD, U/L	31.32 ± 7.56	33.47 ± 9.68	2.19	0.1392
Alkaline phosphatase, mean ± SD, U/L	66.32 ± 32.42	68.73 ± 17.16	0.83	0.4078
Bilirubin, mean ± SD, mg/dL	1.29 ± 1.02	1.54 ± 1.46	-0.63	0.5293

Table 2 summarizes the intraoperative variables of the study population.

**Table 2:** Intraoperative variables of the study cohorts *Significant value (p<0.05); RC: Robotic cholecystectomy; LC: Laparoscopic cholecystectomy; SD: Standard deviation

Parameter	RC (N=91)	LC (N=51)	t-value/χ^2^	P-value
Conversion to open, n (%)	0 (0.0)	8 (15.69)	-	<0.001*
Total ports used, mean ± SD	4.22 ± 0.55	3.85 ± 0.73	3.41	0.0080*
10 mm ports, mean ± SD	1.2 ± 0.42	1.83 ± 0.52	-7.86	<0.001*
8 mm ports, mean ± SD	3.04 ± 0.21	2.5 ± 0.71	6.76	<0.001*
5 mm ports, mean ± SD	1.23 ± 0.43	1.96 ± 0.60	-8.39	<0.001*
Cystic duct (converted from clip to suture), n (%)	18 (19.78)	9 (17.65)	0.10	0.7560
Intraoperative complications, n (%)	9 (9.89)	11 (21.57)	3.68	0.0549
Bile duct injuries, n (%)	0 (0.0)	0 (0.0)	-	-
Adhesions, n (%)	45 (49.45)	51 (100.0)	-	<0.001*

A total of eight patients (15.69%) in the LC group required conversion to open surgery, whereas no conversions were observed in the RC group (p<0.001). In the RC group, the standard port configuration consisted of three 8 mm ports, with an optional 5 mm assistant port, whereas LC was typically performed using two 5 mm and two 10 mm ports. Port utilization differed significantly between the two approaches, with RC procedures requiring a higher average number of ports than LC procedures (4.22 ± 0.55 vs. 3.85 ± 0.73; p=0.0080). In the RC group, 20 patients required five ports and two required six ports due to concomitant procedures. In comparison, additional ports for concomitant procedures were required in five patients in the LC group, of whom three required five ports and two required six ports.

The proportion of patients requiring conversion of cystic duct closure from clip application to suturing was comparable between the two groups. Intraoperative complications occurred less frequently in the RC group than in the LC group; however, the between-group difference was not statistically significant (9.89% vs. 21.57%; p=0.0549). All intraoperative events in both cohorts were classified as Grade II based on the Clavien-Dindo system [14]. No bile duct injuries were observed in either group. Adhesions around the gallbladder were present in 51 (100%) patients undergoing LC, and in 45 (49.45%) patients undergoing RC.

Table 3 summarizes postoperative outcomes with follow-up through 30 days.

**Table 3:** Postoperative outcomes of the study cohorts *Significant value (p<0.05); RC: Robotic cholecystectomy; LC: Laparoscopic cholecystectomy; SD: Standard deviation

Parameter	RC (N=91)	LC (N=51)	t-value/χ^2^	P-value
ICU duration, mean ± SD, hours	15.82 ± 10.76	46.14 ± 19.25	-12.05	<0.001*
White blood cells, mean ± SD, 10^3 ^cells/µL	8.01 ± 3.95	7.37 ± 3.05	1.00	0.3184
Alanine aminotransferase, mean ± SD, U/L	36.41 ± 15.89	37.86 ± 21.50	-0.46	0.6476
Aspartate aminotransferase, mean ± SD, U/L	38.54 ± 26.24	41.18 ± 20.98	-0.62	0.5387
Alkaline phosphatase, mean ± SD, U/L	73.52 ± 29.28	72.94 ± 23.00	0.12	0.9032
Bilirubin, mean ± SD, mg/dL	1.23 ± 0.81	1.32 ± 1.26	-0.52	0.6057
Time to ambulation, mean ± SD, hours	12.80 ± 5.71	28.47 ± 13.05	-9.91	<0.001*
Time to oral intake, mean ± SD, hours	13.31 ± 5.03	25.65 ± 12.23	-8.45	<0.001*
Time to bowel movement, mean ± SD, hours	32.37 ± 9.98	28.94 ± 11.67	1.85	0.0668
Length of hospital stay, mean ± SD, days	2.45 ± 0.78	4.37 ± 1.36	-10.70	<0.001*
Postoperative complications, n (%)	0	0	-	-
Complications within 30 days, n (%)	0	0	-	-
Readmission within 30 days, n (%)	0	0	-	-
30-day mortality, n (%)	0	0	-	-
Pain score, mean ± SD				
Day 0	2.42 ± 1.02	3.37 ± 0.77	-5.79	<0.001*
Day 7	0.71 ± 0.97	1.96 ± 0.89	-7.58	<0.001*
Day 14	0.21 ± 0.57	0.73 ± 0.80	-4.49	<0.001*
Day 30	0.01 ± 0.11	0.12 ± 0.33	-2.91	0.0042*
Return to activities of daily living, mean ± SD, days	4.45 ± 0.92	6.75 ± 1.98	-5.79	<0.001*

Although all patients were admitted to the ICU following surgery, the length of ICU stay differed significantly between groups, with the RC cohort demonstrating a shorter mean duration than the LC cohort (15.82 ± 10.76 vs. 46.14 ± 19.25 hours; p<0.001). Postoperative laboratory parameters, including WBC count, ALT, AST, ALP, and bilirubin levels, were comparable between the groups, with no statistically significant differences observed. The RC group demonstrated significantly faster postoperative recovery than the LC group, with shorter time to ambulation (12.80 ± 5.71 vs. 28.47 ± 13.05 hours; p<0.001), earlier resumption of oral intake (13.31 ± 5.03 vs. 25.65 ± 12.23 hours; p<0.001), earlier return of bowel function (32.37 ± 9.98 vs. 28.94 ± 11.67 hours; p=0.0668), and reduced length of hospital stay (2.45 ± 0.78 vs. 4.37 ± 1.36 days; p<0.001). No postoperative complications, readmissions, or mortality were recorded in either group during the 30-day follow-up period. VAS-based postoperative pain scores were consistently lower in the RC group at 24 hours and on postoperative days seven, 14, and 30, with statistical significance observed at each time point (p<0.001 for 24 hours, day seven, and day 14; p=0.0042 for day 30). In addition, patients undergoing RC returned to activities of daily living significantly earlier than those in the LC group (4.45 ± 0.92 vs. 6.75 ± 1.98 days; p<0.001).

## Discussion

RC has seen a significant increase in adoption for benign surgical procedures in India over the past five years. During this period, several Indian studies have evaluated perioperative outcomes of RC in comparison with LC. To our knowledge, evidence from India comparing RC and LC for AC remains limited, with only a single study directly addressing this comparison [3]. The present study was undertaken to address this gap by comparing perioperative and functional outcomes of RC and LC for AC in an Indian population.

The primary outcomes assessed were intraoperative complications, conversion rate, and length of hospital stay. RC was associated with no conversions and a significantly shorter duration of hospitalization compared with LC, underscoring its potential advantages in the management of AC. Although the intraoperative complication rate was numerically lower in the RC group, the difference narrowly missed statistical significance. Notably, favorable outcomes in the RC cohort were achieved despite a higher proportion of male patients, often associated with more advanced disease at presentation, and a higher preoperative risk profile, with 10 (10.99%) patients classified as ASA physical status IV. Importantly, all intraoperative complications in both groups were categorized as Grade II under the Clavien-Dindo classification and were managed without the need for invasive intervention, potentially indicating robust surgical expertise and protocol-driven perioperative care.

Notably, the LC group required more frequent and prolonged administration of broad-spectrum antibiotics, including meropenem, suggesting a comparatively greater postoperative inflammatory burden. The observed superiority of RC with respect to conversion rates and length of hospital stay may be attributed to the enhanced three-dimensional visualization, superior instrument articulation, tremor filtration, and ergonomic precision afforded by the robotic platform. These technical advantages appear to translate into improved intraoperative control in inflamed and anatomically distorted operative fields. In addition, completion cholecystectomy was achieved in a higher proportion of patients undergoing RC, whereas subtotal cholecystectomy was more frequently required in the LC group. The routine use of fluorescence-guided imaging with indocyanine green in the RC cohort likely facilitated safer dissection and more reliable identification of biliary anatomy, thereby reducing the need for bail-out procedures. Robotic systems have been shown to enhance the performance of minimally invasive surgery by enabling movements analogous to open surgery while preserving the benefits of keyhole access through improved stability, precision, and surgeon ergonomics [15]. These advantages are particularly relevant in AC, where severe inflammation, edema, and dense adhesions substantially increase operative complexity and the risk of conversion and intraoperative complications [16]. Collectively, our findings provide further evidence supporting the expanding role of robotic surgery as an effective and reliable approach for managing complex benign biliary disease in the Indian clinical setting, especially in centers with appropriate expertise and infrastructure.

The existing literature presents heterogeneous and sometimes conflicting evidence regarding the comparative benefits of RC and LC. A 2017 meta-analysis encompassing both elective and emergency procedures reported no significant differences between RC and LC with respect to intraoperative complications, conversion rates, estimated blood loss, or length of hospital stay [17]. Similarly, several studies have suggested that the technical advantages of robotic surgery, particularly enhanced visualization and instrument precision, do not consistently translate into a reduction in intraoperative complication rates [8,9]. In the specific context of AC, however, emerging evidence indicates a potential advantage of RC. Some studies have demonstrated significantly lower conversion rates with RC compared with LC [3,17,18], while others have reported lower intraoperative complication rates without a corresponding reduction in conversion rates [19]. These discrepancies likely reflect variations in patient selection, disease severity, surgeon experience, and institutional practice patterns. Our findings align with studies reporting a reduced conversion rate in patients undergoing RC for AC. While the between-group difference in intraoperative complication rates was not statistically significant, the lower incidence observed in the robotic group suggests that the technical attributes of robotic systems may offer advantages when operating in challenging inflammatory conditions. Collectively, these data suggest that while RC may not universally outperform LC across all outcomes, it may confer specific advantages in selected patients with AC.

Ray and Dhar [5] reported that LC was associated with significantly higher postoperative opioid requirements compared with RC, and suggested that reduced postoperative pain following RC may facilitate earlier ambulation. Similarly, a comparative study of single-incision versus conventional three-port LC demonstrated reduced postoperative pain and earlier ambulation in the single-incision group [20]. Several Indian studies have consistently reported lower postoperative pain scores following RC compared with LC [3,4,6], with one study noting that reduced pain translated into earlier ambulation and a faster return to work [6]. Another long-term analysis suggested that LC may be associated with higher pain severity than RC persisting up to two to seven years postoperatively [21]. Our findings indicate that RC was associated with consistently lower postoperative pain scores throughout the first 30 postoperative days and facilitated earlier ambulation relative to LC. These findings are concordant with existing evidence indicating that minimally invasive techniques such as RC and single-site laparoscopy are associated with reduced postoperative pain and accelerated functional recovery [20]. The reduction in pain likely contributes to earlier mobilization, which may further facilitate recovery. In addition, we observed earlier resumption of oral intake and earlier return of bowel function in the RC group, which may partly explain the significantly faster return to activities of daily living observed in this cohort. Contrasting evidence does exist. One study comparing single-incision RC with single-incision LC reported a longer time to return to independent daily activities in the RC group, with no difference in conversion rates and a longer duration of narcotic use among RC patients [7]. These discrepancies may reflect differences in surgical technique, patient selection, institutional protocols, and surgeon experience. Overall, our findings suggest that the advantages of RC, particularly reduced postoperative pain and earlier functional recovery are clinically meaningful. In our experience, these benefits translate into a lower recovery burden for patients and enable an earlier return to routine activities and productivity, supporting the role of RC as an effective approach for managing AC in appropriately selected patients.

No bile duct injuries were observed in either group. This finding is consistent with prior studies reporting comparable or lower bile duct injury rates with RC compared with LC [22-24]. Although conversion of the cystic duct closure technique from clip application to suturing was required in a subset of cases, the frequency of conversion did not differ significantly between the two groups. Furthermore, no postoperative complications or readmissions were recorded in either group during the study follow-up period.

Strengths and limitations

This study has several notable strengths. It focuses exclusively on complex AC (Tokyo Grade II-III), enabling disease-specific interpretation of outcomes. All procedures were performed by a single experienced surgeon beyond the learning curve for both laparoscopic and robotic techniques, thereby minimizing operator-related variability. The use of a prospectively maintained database and standardized perioperative protocols further enhanced data reliability. Importantly, the study assessed both perioperative and patient-centered recovery outcomes, including postoperative pain, functional recovery, and return to activities of daily living, domains that remain underreported in the existing literature.

Nevertheless, several limitations should be acknowledged. The retrospective, single-center nature of the study limits causal interpretation and may constrain the generalizability of the results. Moreover, non-randomized allocation of surgical approach and unequal group sizes introduce the potential for selection bias. In addition, outcomes may not be directly applicable to centers with limited experience in robotic surgery. Finally, follow-up was restricted to 30 days, precluding evaluation of long-term outcomes, durability of benefits, and cost-effectiveness.

## Conclusions

This study demonstrates that RC confers meaningful advantages over LC, particularly in complex cases of AC as defined by the Tokyo Guidelines, when performed by surgeons experienced in both techniques. RC was associated with significantly lower conversion rates, shorter ICU and overall hospital stays, reduced postoperative pain, and accelerated functional recovery, including earlier independent ambulation, resumption of oral intake, and return to activities of daily living. These findings suggest that the technical advantages of robotic surgery, enhanced visualization, superior instrument dexterity, and greater precision, translate into tangible clinical benefits and an improved patient experience in complex inflammatory biliary disease. Accordingly, RC should be strongly considered as a preferred surgical approach in selected cases of AC where appropriate expertise and infrastructure are available. The favorable outcomes observed in this study support the broader, evidence-guided adoption of robotic surgery to optimize recovery and perioperative outcomes. In light of the study design, these findings warrant cautious interpretation, and further validation through rigorously designed, large-scale prospective studies with extended follow-up across varied clinical environments is necessary.
